# Anti-CD44 antibodies inhibit both mTORC1 and mTORC2: a new rationale supporting CD44-induced AML differentiation therapy

**DOI:** 10.1038/leu.2016.221

**Published:** 2016-09-13

**Authors:** S Z Gadhoum, N Y Madhoun, A F Abuelela, J S Merzaban

**Affiliations:** 1 Biological and Environmental Sciences and Engineering Division, King Abdullah University of Science and Technology (KAUST), Thuwal, Saudi Arabia

Acute myeloid leukemia (AML) is a heterogeneous disease characterized by a blockage in the differentiation of myeloid cells at different stages of maturity and by an increase in their proliferation. Despite important advances in understanding the pathophysiology of AML, therapeutic approaches have not significantly improved patient survival with the exception of ATRA (all-trans retinoic acid) for acute promyelocytic leukemia (APL),^1^ prompting scientists to search for differentiating agents that could be used in the treatment of all AML subtypes.

Triggering CD44, using monoclonal antibodies (mAbs), is effective at inducing the differentiation and inhibiting the proliferation of many AML subtypes.^2, 3^ CD44 is a transmembrane glycoprotein expressed on both normal and leukemic cells (Supplementary Figure 1), and implicated in multiple functions including proliferation, differentiation, apoptosis and homing to the bone marrow (BM).^4, 5, 6^ Despite current knowledge about CD44 signaling, the molecular mechanisms involved in inhibiting the proliferation and inducing differentiation of AML are not fully understood. The PI3K/Akt/mTOR (mammalian target of rapamycin) pathway, frequently dysregulated in AML,^7, 8^ has not previously been investigated in the context of CD44-signaling in AML. The activation of the Akt signaling pathway results in the loss of control of cell growth and in cancer cell death.^9^ Because the PI3K/Akt/mTOR pathway is also implicated in sensitivity and resistance to therapy, its blockade is an attractive approach for cancer treatment.

To explore the effect of anti-CD44-mAbs on PI3K/Akt/mTOR pathway, we used AML cell lines representing different subtypes: HL60, THP-1 and KG1a. As shown in Figure 1a, A3D8 treatment induced a considerable decrease in the expression of p(phosphorylated)-mTOR on Ser2481, an autophosphorylation event reflecting the catalytic activity of this serine/threonine kinase,^10^ in all cell lines tested as early as 5 min that continued to 24 h after treatment, which did not appear to be related to changes in total mTOR expression. Since PI3K and mTOR pathways are suggested to be independently involved in AML cell proliferation, simultaneously blocking both pathways versus only one, should more effectively inhibit the proliferation of leukemic cells.^11^ We also found that anti-CD44 treatment strongly reduced p-Akt on Thr308, a downstream effector of PI3K in all cell lines (Figure 1a). Similarly, expression of p-mTOR on Ser2481 and p-Akt on Thr308 decreased considerably when primary leukemic blasts (from patients newly diagnosed with AML) were treated with A3D8 (Figure 1b), suggesting that anti-CD44 ligation alters the PI3K pathway in AML cells upstream of mTOR. In contrast, no significant change in the expression of p-mTOR on Ser2481 or p-Akt on Thr308 was observed following A3D8 treatment of normal CD34^+^ BM cells (Figure 1b). This result is in line with previous work reporting that CD44 triggering does not affect the proliferation of these cells.^2^ These results confirm that the anti-CD44-mAbs have specificity towards leukemic cells over normal CD34^+^ cells and this provides a strong argument for the use of CD44 receptor activation as an antileukemic target. Given that all the AML cells tested showed very similar responses to A3D8 treatment, we chose to focus on HL60 cells for most of the subsequent experiments.

Recent work from our lab identified that CD44-mAbs recognize their antigen with various affinities that are dependent on factors such as cell type and glycosylation status,^12^ and brings forth the possibility to consider a variety of CD44-mAb clones in such studies. In order to determine if the observed effect on p-mTOR was specific to the A3D8-mAb triggering, we tested two additional anti-CD44-mAbs that label CD44 (Supplementary Figure 3, left panel): J173 and Hermes-3. J173 is known as a ‘non-relevant antibody' incapable of inducing cell growth inhibition or differentiation of AML cells^3^ while Hermes-3 is an antibody that induces differentiation of HL60 cells (Supplementary Figure 3, middle panel). Hermes-3 inhibited p-mTOR on Ser2481 similar to A3D8 while J173 did not (Supplementary Figure 3, right panel), suggesting that the observed decrease in p-mTOR is, in fact, due to the activating antibody treatment and not a consequence of non-specific binding of the antibody to an Fc receptor. More interestingly, it correlates with the inhibition of p-mTOR to the phenotypic changes induced by the anti-CD44-mAbs (that is differentiation) (Supplementary Figure 3, middle panel).

To investigate whether activating CD44-mAbs would be effective at inhibiting mTOR activity *in vivo*, we used a recently described mouse model of leukemia, established using HL60 cells adoptively transferred into NOD/SCID mice (Supplementary Figure 4), and confirmed a significant decrease in p-mTOR expression in the human CD45^+^ fraction of the BM of mice that were treated with CD44-mAbs compared with isotype-matched control (Supplementary Figure 4).

Since mTOR is part of two exclusive multiprotein complexes, mTORC1 and mTORC2,^10, 13^ we sought to investigate which of these complexes is affected by anti-CD44-mAb treatment. p70S6K is a major effector of activated mTORC1 complexes that phosphorylates and activates several downstream effectors, including the S6 ribosomal protein. We observed that treatment with A3D8 drastically decreased p-p70S6K on Thr389 (a direct target of mTORC1) (Figure 1c), which is correlated with a decrease in p-mTOR proteins (Figure 1). The mTORC2 complex controls cell proliferation and cell survival through the phosphorylation of Akt on Ser473^(ref. 13)^ and is found to be high in many types of AML blasts.^14^ Treatment of AML cell lines with A3D8 significantly decreased p-Akt on Ser473 as a consequence of the upstream inhibition of mTORC2 (Figure 1c). Altogether, these data show that the CD44 triggering using A3D8-mAb inhibits both mTORC1 and mTORC2 complexes. This is a significant achievement since rapamycin (a specific blocker of mTOR activity) was only modestly successful in leukemic clinical trials, largely due to its higher specificity towards mTORC1 over mTORC2 allowing it to activate Akt via phosphorylation of Ser473.^13, 15^

A key functional cellular response downstream of the mTOR pathway is the regulation of the initiation of cap-dependent mRNA translation, which occurs primarily via regulation of the 4E-BP1 repressor of mRNA translation.^13^ Phosphorylation of 4E-BP1 is essential for the deactivation of the protein and its subsequent dissociation from eIF4E, which must take place for cap-dependent mRNA translation to proceed. One of the limitations of rapamycin is its failure to inhibit oncogenic protein translation.^13^ Analysis of the expression of p-4E-BP1 on Ser65 revealed significant baseline levels in AML cells that was inhibited following treatment particularly at 24 h, thereby implying the inhibition of protein translation in these cells (Figure 1c).

Survivin is an inhibitor of apoptosis that is overexpressed in hematological malignancies including AML and its expression is a marker of poor AML prognosis.^16^ Because survivin is a transcriptional target of CD44 signaling,^17^ and its expression is regulated by multiple signaling pathways, including the PI3K pathway,^16^ we investigated the effect of anti-CD44-mAbs on its expression in AML cells and found that it was most significantly decreased at 24 h (Figure 2a) following treatment, although a decrease was apparent at earlier time points (Supplementary Methods).

Akt regulates the activity of multiple targets including the FOXO proteins, particularly FOXO3 transcription factor, which regulates the transcription of genes involved in multiple cellular functions such as proliferation, cell survival and differentiation.^9, 18^ FOXO3 is the only known FOXO protein expressed in AML blasts^18^ and is associated with poor prognosis. When Akt is inactive, FOXOs are underphosphorylated and are localized to the nucleus where they regulate the transcription of genes involved in apoptosis and cell cycle arrest. When phosphorylated, FOXOs translocate from the nucleus to the cytoplasm where they are targeted for proteasome degradation. The Akt/FOXO3 pathway has been recently found to play an important role in maintaining the blockage of differentiation in human myeloid cells and its inhibition can thus be targeted for leukemia therapy.^19^ As shown in Figure 2a, anti-CD44 ligation strongly inhibited p-FOXO3a on Ser253 in agreement with the observed upstream inhibition of p-Akt (Figure 1).

Since the subcellular localization of FOXO3a is closely correlated with its activity,^18^ we used confocal microscopy to observe its distribution. As shown in Figure 2b, prior to treatment, FOXO3a was mainly expressed in the cytoplasm whereas following A3D8 treatment its expression translocated into the nucleus, suggesting that there is a precedence for underphosphorylated FOXO3a. Z-stack (Supplementary Videos 1-8) and colocalization (Figure 2c) analysis showed an increase in the expression of FOXO3a protein in the nucleus.

The mTOR pathway is constitutively activated in many leukemia subtypes and it has been shown that its inhibition can induce both anti-proliferative as well as pro-differentiation effects.^7, 20^ To investigate the implication of the observed downregulation of the PI3K/Akt/mTOR pathway in the inhibition of proliferation of AML cells, HL60 cells were treated with either rapamycin or LY294002 (a potent PI3K inhibitor) and analyzed for proliferation and for the effect on p-mTOR and p-Akt. Either inhibitor alone, rapamycin or LY294002, induced a significant decrease in p-mTOR (Supplementary Figure 5A), which was associated with a significant inhibition of proliferation (Supplementary Figure 5B). The fact that rapamycin is a specific mTOR inhibitor suggests that the mTOR inhibition induced by rapamycin is sufficient for the inhibition of proliferation. A3D8 treatment induced a similar inhibition of proliferation (Supplementary Figure 5B) accompanied by a significant decrease in p-mTOR expression (Supplementary Figure 5A) that is similar to rapamycin and LY294002 alone, suggesting that the mTOR inhibition by anti-CD44-mAbs might be involved in the observed inhibition of proliferation. To establish whether these specific inhibitors enhanced the inhibition of proliferation induced via CD44 ligation, rapamycin or LY294002 or both were added along with A3D8. The concomitant use of A3D8 with rapamycin did not further inhibit proliferation of HL60 cells compared with A3D8 alone (Supplementary Figure 5B). This was confirmed by the similar decrease in p-mTOR expression when cells were treated with A3D8 alone or in the presence of rapamycin and/or LY294002 (Supplementary Figure 5A). Interestingly, no additive effect on the inhibition of proliferation was observed when A3D8 was used in conjunction with the PI3K inhibitor compared with A3D8 alone, despite the stronger inhibition of p-Akt on Thr308 when LY294002 was used along with A3D8 (Supplementary Figure 5A).

The mechanisms through which CD44 ligation inhibits mTOR are yet to be elucidated. Although LY290004 inhibited Akt phosphorylation on Thr308 as well as the cell proliferation, it did not inhibit mTOR phosphorylation as significantly as A3D8. This suggests that if PI3K inhibition is involved in the mTOR inhibition induced by the anti-CD44-mAb, it is not the only pathway responsible for the inhibition of mTOR phosphorylation *via* CD44. Several signaling pathways involved in myeloid proliferation and differentiation act downstream of CD44-receptor ligation such as the mitogen-activated protein kinases (MAPK), including extracellular signal regulated kinase 1 and 2 (ERK1/2) and src family kinases (SFKs), including Lyn, Fgr and Hck.^21^ An attractive candidate for the cross-talk between CD44 and mTOR is the non-receptor tyrosine kinase Syk, which has emerged as a critical regulator of mTOR in AML blasts.^22^

KG1a is a leukemic cell line whose inhibition of proliferation but not differentiation (contrary to HL60 and THP-1 cells) is induced by anti-CD44-mAb treatment^3^ (Supplementary Figure 6). Interestingly, anti-CD44-mAb treatment of these cells also resulted in the inhibition of p-mTOR. Moreover, treatment of HL60 cells with rapamycin inhibited their proliferation but did not result in granulocytic differentiation (Supplementary Figure 6 legend). Together these findings suggest that mTOR inhibition is not sufficient to induce differentiation of AML cells.

In summary we provide compelling evidence that the inhibition of proliferation, and in some cases the induction of differentiation, of AML cells induced by anti-CD44-mAb treatment is accompanied by a marked decrease in the phosphorylation of the mTORC1 and mTORC2 complexes, which is strongly correlated with the inhibition of the PI3K/Akt pathway. Since perturbations in the PI3K and mTOR pathways are commonly observed in leukemia, blocking two major players of the PI3K/Akt/mTOR pathway^11, 23^ along with inhibiting protein translation, reinforces the idea of using anti-CD44-mAbs in therapeutic strategies for leukemia.

## Figures and Tables

**Figure 1:**
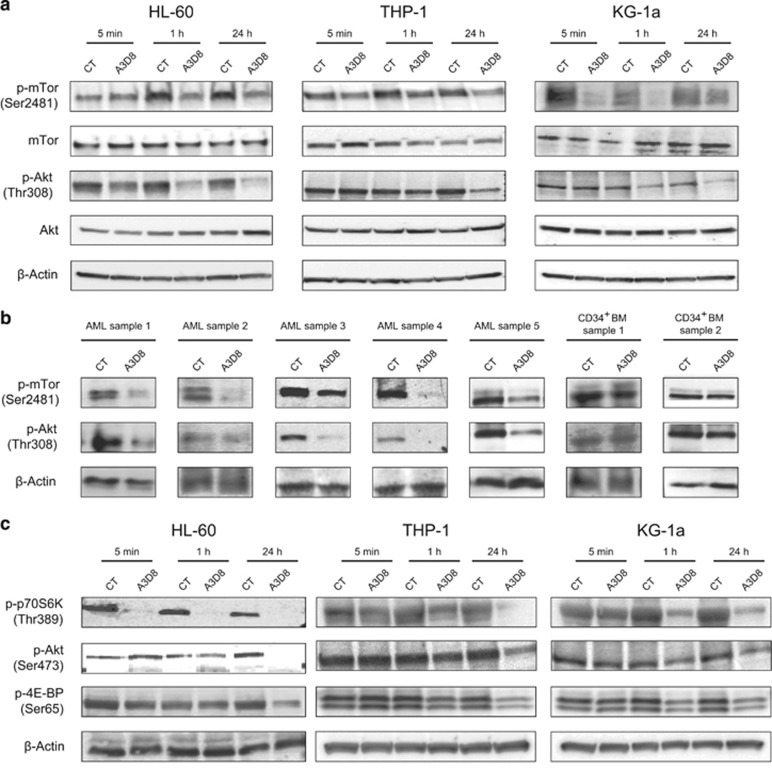
Anti-CD44 treatment strongly inhibits the PI3K/Akt/mTOR pathway in AML cells. (**a**) HL60, KG1a and THP-1 cells were cultured with mIgG_1_-mAb (CT) or with A3D8 (both at 2.5 μg/mL) for the indicated time points and cell lysates (*n*=5 for HL60, *n*=3 for THP-1 and KG1a) were then subjected to western blot analysis using antibodies against p-mTOR (Ser2481), total mTOR, p-Akt (Thr308) and total Akt. Note that this decrease was directly correlated to the dose of anti-CD44-mAb used, suggesting that this effect was specific to the anti-CD44-mAb treatment (Supplementary Figure 2) (**b**) Primary blast cells isolated from patients with newly diagnosed or relapsed AML and CD34^+^ cells isolated from healthy BM donors were cultured with mIgG_1_-mAb (CT) or with A3D8 for 1 h (patient sample 1) or 24 h (all other samples) and cell lysates were subsequently subjected to western blot analysis using antibodies against p-mTOR (Ser2481) and p-Akt (Thr308). Anti-β-actin was used as a loading control for all experiments. Representative data is shown for five AML patients and for two CD34^+^ normal BM cells. (**c**) HL60, KG1a and THP-1 cells were cultured with mIgG_1_ (CT) or with A3D8 (2.5 μg/mL) and cell lysates were subsequently subjected to western blot analysis using antibodies against p-p70S6K (Thr389), p-Akt (Ser473) and p-4E-BP (Ser65). Anti-β-actin was used as a loading control. One representative experiment of *n*=3 is shown.

**Figure 2:**
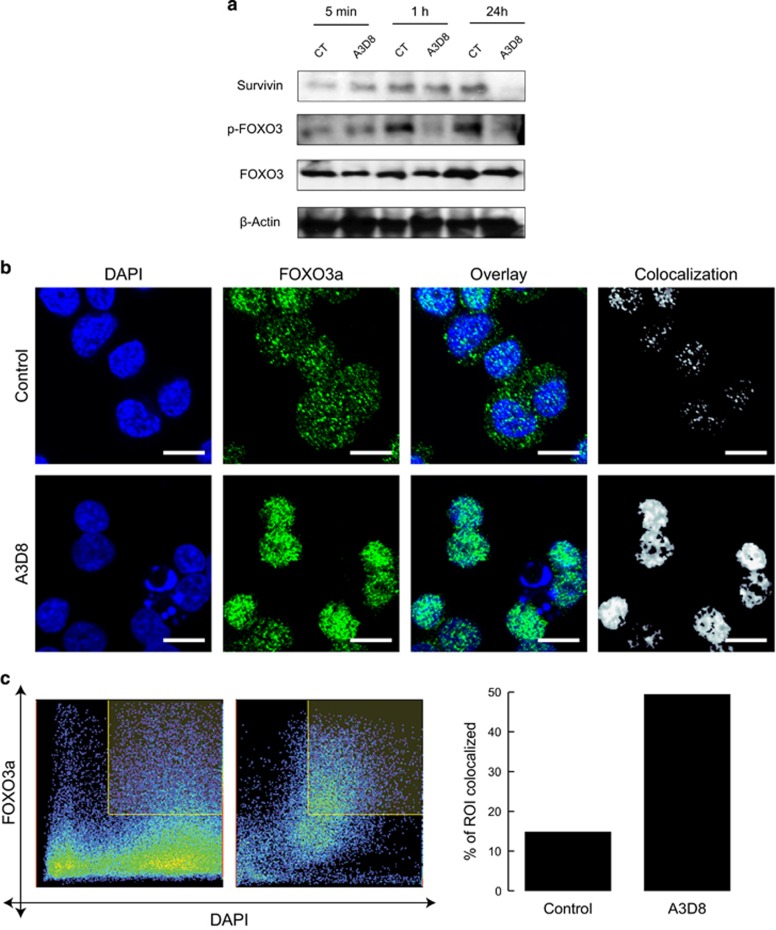
Significant anti-leukemic effects result following treatment of AML cells with anti-CD44-mAbs, which are coincident with reduction in markers of poor disease prognosis. (**a**) HL60 cells were cultured in presence of mIgG_1_ (CT) or of A3D8 (at 2.5 μg/mL) for the indicated times and subsequently cell lysates were subjected to western blot analysis using antibodies against survivin, p-FOXO3a (Ser253) and total FOXO3a protein. β-actin was used as a loading control. (**b**) A3D8 treatment leads to movement of FOXO3a out of the cytoplasm and into the nucleus. mIgG_1_-treated (CT) (upper panels) and A3D8-treated (lower panels). HL60 cells were fixed, permeabilized, and stained for total FOXO3a (green) and DAPI DNA nuclear stain (blue) prior to confocal fluorescence imaging. Colocalization mask of the green and blue channels is represented in white. One representative experiment of *n*=3 is shown. Scale bar, 10 μm. (**c**) HL-60 cells were treated with IgG_1_ (control) or A3D8 and immunofluorescence staining of FOXO3a was performed as well as nuclear staining using DAPI. Colocalization was analyzed from (**b**) with the nuclei as regions of interest (ROI). (Left) Dot plot of the distribution of pixel intensities within the ROI shows higher correlation between FOXO3a signal and DAPI signal compared with the control. (Right) The percentage of colocalized area was calculated in the ROI and showed higher colocalization in A3D8-treated cells (49.4%) compared with the control (14.8%). Z-stack analysis was also performed to show that the expression of FOXO3a protein predominated in the nucleus as illustrated in Supplementary Videos 1-8.

## Supplementary Material

Supplementary Informations
