# Supplementary material for: Anti-CD44 antibodies inhibit both mTORC1 and mTORC2: a new rationale supporting CD44-induced AML differentiation therapy
Source: Leukemia. 2016 Sep 13;30(12):2397–401. doi: 10.1038/leu.2016.221 (PMC5155032; doi:10.1038/leu.2016.221)
Supplement: Supplementary Informations [file leu2016221x1.docx]

**Anti-CD44 antibodies inhibit both mTORC1 and mTORC2: a new rationale supporting CD44-induced AML differentiation therapy**

Samah Z. Gadhoum, Nour Y. Madhoun, Ayman F. Abuelela and Jasmeen S. Merzaban*

**SUPPLEMENTARY INFORMATION**

**MATERIALS AND METHODS**

**Cells.** HL60 (AML FAB M2; AML, not otherwise specified, AML with maturation),[^1^](#_ENREF_1)^,^ [^2^](#_ENREF_2) KG1a (AML FAB M0; AML, not otherwise specified, undifferentiated AML) and THP-1 (AML FAB M4/5; AML with genetic abnormalities, t(9;11)(p21;q23) leading to *MLL-MLLT3* (*MLL-AF9*) fusion gene)[^3^](#_ENREF_3)^,^ [^4^](#_ENREF_4) cells were purchased from ATCC (Manassas, VA, USA). AML cell lines were exponentially grown in RPMI 1640 medium (Gibco, Grand Island, NY, USA) supplemented with 10% heat-inactivated fetal bovine serum (FBS), 100U/ml penicillin, and 100μg/ml streptomycin (Gibco/Invitrogen, Grand Island, NY, USA) in a 5%CO_2_ humidified atmosphere at 37°C. Leukemic samples from patients with newly diagnosed/relapsed AML [patient 1 (newly diagnosed): AML with genetic abnormalities, t(6;11)(q27;q13), >80% myeloid blasts; patient 2 (relapsed): AML with genetic abnormalities, t(8;16)(p11;p13), 80% myeloid blasts; patient 3 (newly diagnosed): AML,, patient clinical report was incomplete at time of collection; >50% myeloid blasts; patient 4 (relapsed): AML with genetic abnormalities, t(6;11)(q27;q13), >80% myeloid blasts; patient 5 (relapsed): AML with genetic abnormalities, t(7;13?)(q22;q14), 95% monoblast/myeloid blasts] and normal CD34^+^ cells from healthy allogenic donors were purchased from ALLCells (Alameda, CA, USA).

**Cell Treatment.** Cells were harvested, resuspended in fresh medium without FBS, seeded at a density of 1×10^6^ cells/mL, and treated with anti-CD44-mAb A3D8 (mouse IgG_1_, Sigma Aldrich), with J173 (mouse IgG_1_, Beckman Coulter), Hermes-3 (mouse IgG_2a_, hybridoma ATCC) or with an a mIgG_1_ (BD Pharmingen) isotype control, all at 2.5 μg/mL (unless otherwise stated) for up to 72h. Rapamycin and LY294002 were purchased from Sigma (St Louis, MO, USA). Stock solutions of these agents were subsequently diluted with serum-free RPMI-1640 medium prior to use at a final concentration of 10 μM for LY294002 and 20 nM for rapamycin.

**Cell proliferation and cell differentiation assessment**. The mTOR pathway is constitutively activated in many leukemias and it has been shown that its inhibition can induce both anti-proliferative as well as pro-differentiation effects.^[5-8](#_ENREF_5)^ To investigate the implication of the down-regulation of the PI3K/Akt/mTOR pathway observed in the inhibition of proliferation of AML cells, HL60 cells were treated with either rapamycin or LY294002 (a potent PI3K inhibitor) and analyzed for proliferation and for the effect on p-mTOR and p-Akt. Triplicates of treated and untreated HL60 cells were incubated at 37^0^C for 72 h then counted using trypan blue exclusion. Myeloid differentiation was evaluated according to the increased expression of myeloid differentiation antigens, CD11b (for granulocytic and monocytic differentiation), CD14 (for monocytic differentiation), and CD15 (for granulocytic differentiation) using flow cytometry as described below.

**Giemsa Staining**. HL60 and THP-1 cells control or treated with A3D8 for 72 h were washed twice with PBS/2%FBS. Cells were cytocentrifuged at 500RPM for 5 min, fixed with methanol for 15 min and allowed to dry completely and then stained with Giemsa staining (Sigma-Aldrich), a solution of 1:20 diluted in PBS (6.8 pH), for 4 h. After staining, the smears were rinsed quickly with distilled water and allowed to air dry before mounting and visualizing under a microscope.

**Flow cytometry.** Cells (2 × 10^5^) were washed with PBS/2% FBS and incubated with the fluorochrome conjugated or unconjugated primary mAbs or the corresponding isotype control then washed 3 times in PBS/2% FBS, incubated when needed with the appropriate secondary fluorochrome-conjugated antibody. After 3 washes, fluorescence intensity was determined using a (Becton Dickinson FACSCanto Flow cytometer) previously described.^[9](#_ENREF_9), [10](#_ENREF_10)^

**Cell lysis and Western blot.** Cells were washed in 1X PBS, lysed in lysis buffer [1% Triton X-100, 50mM Tris-base (pH 8.0), 150mM NaCl, 1mM PMSF, cOmplete protease inhibitor cocktail (Roche, Indianapolis, USA)] and mechanically homogenized. Equal amounts of total proteins (20mg) were boiled for 5 min in SDS polyacrylamide gel electrophoresis (PAGE) sample buffer, loaded onto 4–20% gradient SDS-PAGE, fractionated by electrophoresis for 1.5 h at 100V, and transferred onto PVDF membranes. The membrane was blocked for 4 h or overnight at 4^o^C in blocking solution [5% nonfat dry milk dissolved in 1% Tween20-tris-buffered saline (TTBS)] to inhibit nonspecific binding and then incubated with primary antibodies against mTOR (7C10), phospho-mTOR (Ser2481 [rabbit polyclonal cat#2974L]), phospho-Akt (Ser473 [98H9L8] and Thr308 [B18H12L21]), total Akt (C67E7), p70S6K (49D7), phospho-p70S6K (108D2), FOXO3a (75D8), phospho-FOXO3a (rabbit polyclonal cat#9466), phospho-4E-BP (236B4), total 4E-BP (3M11) or survivin (71G4B7). All antibodies were purchased from Cell Signaling (MA, USA). β-actin monoclonal antibody was used as a loading control (from Becton Dickinson, USA). After incubation, the membranes were washed three times in TBS/Tween and incubated at room temperature for 1 h with HRP-conjugated secondary antibody diluted (1:10000) in TBS-Tween. Immunoreactive proteins were detected using SuperSignal West Pico Chemiluminescent Substrate (Pierce). Band intensities were quantified by densitometric analysis using Image J software. The test proteins (A3D8 treated) and control proteins (mIgG_1_) were then divided by the corresponding β-actin level in the sample. Subsequently, the sample was then normalized using the control protein (mIgG1 isotype treated). Band intensities for survivin blot in **Fig. 2A** were as follows from left to right: 1, 0.8, 1, 0.5, 1, 0.01.

**Immunoprecipitation.** Following cell lysis, 100–200μg protein samples were precleared with protein G-agarose and then incubated with anti-p-mTOR antibodies. The immunoprecipitates were recovered 24 h later and subjected to Western blot analysis along with 20–50 μg of total whole cell lysate.

**Confocal microscopy.** FOXO3 is the only known FOXO protein expressed in AML blasts^[11](#_ENREF_11)^ and is associated with poor prognosis.^[12](#_ENREF_12)^ A3D8-treated (for 24 h) and mIgG_1_-treated (control) HL60 cells were washed twice in PBS and fixed with PBS containing 4% paraformaldehyde at room temperature. Cells were then permeabilized and incubated with a blocking buffer solution containing 0.03% Triton X-100 and 5% goat serum for 1 h at room temperature to avoid any non-specific binding. Cells were incubated with the primary antibody, rabbit anti-FOXO3a, at a concentration of 1μg/mL overnight at 4°C. After two washes with PBS, cells were incubated with Alexa Fluor 488-conjugated secondary antibody for 2 h at room temperature. Nuclei staining and cell mounting was performed using ProLong Gold Antifade Reagent with DAPI (Molecular Probes). Confocal images were obtained using a LSM 710 laser scanning microscope (Carl Zeiss) and ZEN 2009 software (Carl Zeiss) using 63X oil immersion objective. Colocalization analysis was performed using Imaris software (Bitplane) where the nuclei were set as regions of interest (ROI) using the signal from the DAPI detector channel for subsequent quantification of colocalization. FoxO3 signal was thresholded at the level of signal intensity in the cytoplasm of the control sample and applied to both control and treated sample. Colocalized FoxO3 signal with the ROI was calculated and represented as white pixels in the colocalization result image. Pixel intensity distribution was represented as a dot plot of the signal from the DAPI channel versus the signal from the AF488 channel. Percentage of ROI colocalized was calculated as a quantifier of colocaization in A3D8 sample compared to the control. For the analysis of cellular 3D distribution of FOXO3 protein in A3D8-treated cells, a z-stack of at least 40 sections were obtained with optical section separation (z-interval) of 0.4 um. Rendering of the z-stack images and 3D reconstruction was done using Imaris software (Bitplane).

**Preparation of CD44 mAbs from Hermes-3 hybridoma supernatant.**

The Hermes-3 hybridoma cell line (ATCC), was grown in RPMI 1640 medium containing 10% FBS, 0.05 mM 2-mercaptoethanol, and 1% ampicillin and streptomycin (incubated at 37^o^C with 5% CO_2_). Cells were passed every 2-3 days and then supernatant was collected and incubated with the appropriate volume of protein A agarose for 4 hours at 4^o^C and then subjected to affinity chromatography. The resin was washed with 50 mM Tris-HCl, pH 7.4; 150 mM NaCl, and the desired antibody was eluted with 50 mM Glycine-HCl, pH 2.7. The eluate was then neutralized with 1 M Tris-HCl, pH 8.0; 1.5 M NaCl. PBS buffer exchange was performed following neutralization using amicon columns and the concentration was measured using nanodrop.

**Xenotransplantation of human leukemic cells and in vivo treatment of mice with anti-CD44 antibody.** Animals were used in accordance with a protocol reviewed and approved by the Institutional Animal Care and User Ethical Committee of at Washington Biotechnology. 20 NOD/SCID mice were obtained from Charles River Laboratories. Mice were housed in sterile conditions with filter-topped cages, autoclaved bedding and fed with irradiated food and acidified water. The NOD-SCID repopulation assay by intravenous injection of human HL60 cells was performed as described in a recent study.^[13](#_ENREF_13), [14](#_ENREF_14)^ Briefly, adult female mice (6**–**8 weeks old) were treated with 20 mg/kg busulfan (Sigma) by intraperitoneal administration 24 h before (Day -1) injection of HL60 cells. HL60 cells were washed twice in phosphate buffered saline (PBS) and cleared of aggregates and debris using a 0.2-mm cell filter, and suspended in PBS at a final concentration of 0.2 million cells per 100 μl of PBS per NOD/SCID mouse for intravenous injection on Day 0 via tail vein. On day 10, mice were randomly assigned to 2 experiments: Experiment 1 was comprised of mice that were treated with either anti-CD44 antibody (Hermes-3) or the isotype control (mIgG2a) at a dosage of 200 mg/injection three times a week for one week; Experiment 2 was comprised of mice that were treated similarly to Experiment 1 except for a duration of 2 weeks. The antibodies were prepared in PBS and intraperitoneally injected at a dosage of 200 **m**g per injection three times a week. Daily monitoring of mice for symptoms of disease (ruffled coat, hunched back, weakness and reduced motility) was assessed. Mice were sacrificed at the end of each experiment and whole blood, femurs and tibias were harvested, processed as single cell suspensions and mononuclear cells were isolated by Ficoll-Paque gradient and preserved in liquid nitrogen.

On the day of analysis, bone marrow cells were thawed and prepared for flow cytometric analysis. Cells were first fixed using 4% paraformaldehyde in PBS and then permeabilized using ice-cold methanol for 1 hour. Samples were then blocked with 5% goat serum in PBS for 1hr and stained with primary antibody against phospho-mTOR (Ser2481) rabbit polyclonal [Thermofisher], washed 3 times with PBS/1% BSA and then incubated with secondary FITC-conjugated antibody, washed and analyzed using flow cytometry. The cells were also stained with a mouse antibody specific to human CD45 in order to aid in the identification of human cells in the samples. In addition, differentiation was assessed following *in vivo* treatment with anti-CD44 antibody by analyzing CD14 mAb and CD15 of the human CD45^+^ fraction of the bone marrow. Statistical significance was determined using a two-tailed, two-sample equal variance Student *t*-test.

**Statistical Analysis.** Data are expressed as the means ±S.E. Statistical significance of differences between means was determined by Student *t*-test. Statistical significance was defined as *p* < 0.05.

**SUPPLEMENTARY FIGURES**

**SUPPLEMENTARY FIGURE 1:** **CD44 is highly expressed on normal and diseased primary bone marrow cells.** A representative histogram (of n = 5) showing CD44 expression by flow cytometric analysis of AML blast cells (representing >80% CD34^+^ blast cells) compared to normal CD34^+^ bone marrow cells. This confirms results from previous reports.^[15](#_ENREF_15)^

**SUPPLEMENTARY FIGURE 2: Dose dependent decrease of mTOR phosphorylation in response to increasing A3D8 concentration.** Western blot analysis was used to analyze p-mTOR after a 1h treatment with increasing doses of A3D8 mAb (0, 0.2, 0.5, 1, 2.5 μg/mL) as indicated in the figure. This blot is representative of n = 3 independent experiments.

**SUPPLEMENTARY FIGURE 3: The decrease in mTOR phosphorylation is due to the use of activating CD44 antibody treatments and not a consequence of non-specific binding.** Representative histogram showing flow cytometric analysis of HL60 cells stained with CD44-mAbs: J173, A3D8 or Hermes-3 (*left panel*; n = 5). HL60 cells were treated with mIgG_1_ (CT), A3D8, Hermes-3 or J173 for 72 h and flow cytometric analysis for the expression of the differentiation markers, CD11b and CD15, were assessed. The bar graphs (*middle panel*) show that treatment with A3D8 and Hermes-3 but not with J173, effectively induced differentiation of HL60 leukemic cells. This bar graph is representative of *n = 3* independent experiments. To determine the effect of these CD44-mAbs on the phosphorylation of mTOR and total mTOR expression, western blot analysis 24 h following treatment was performed (*right panel; n = 2*). β-actin was used as a loading control. The western blot shows that A3D8 and Hermes-3 are able to inhibit the expression of p-mTOR in HL60 cells while J173 was not.

**SUPPLEMENTARY FIGURE 4: Activating CD44-mAbs are effective at inhibiting mTOR activity in a mouse model of leukemia.** CD44-mAbs (anti-CD44) or isotype-matched controls (IgG) were injected (3 times/week) into NOD/SCID mice that had been adoptively transplanted with HL60 cells 10 days prior as indicated in the figure (*upper panel*). At the conclusion of each study, mice were euthanized and BM was harvested, single cell suspensions were prepared, double stained for human CD45 mAb and phosphorylated mTOR and analyzed by flow cytometry as shown in the schema (*lower left panel*). Analysis of the results from these 2 experiments showed a consistent decrease of mTOR phosphorylation in CD45^+^ cells of the BM isolated from mice that received CD44-mAbs compared to isotype control (*lower right panel*). There were n = 4-5 mice per treatment group. For each mouse within a group, the mean fluorescence intensity (MFI) is presented as the ratio of the fluorescence intensity to isotype control and is representative as the average of triplicates. The value presented for each group is an average of the individual means (n = 4-5 mice per group) MFI ± SEM *P <* 0.05 for experiment 1 (black bars) and *P <* 0.01 for experiment 2 (white bars) between IgG control and anti-CD44.

**SUPPLEMENTARY FIGURE 5: mTOR inhibition is tightly correlated with the inhibition of proliferation of AML cells. (A)** Cells were seeded at 0.5 x 10^6^ cells/mL and were treated with mIgG_1_ (CT; 2.5 μg/mL), A3D8 (2.5 μg/mL), LY294002 (10 μM), rapamycin (20 nM) or a combination as indicated in the figure. Cell lysates were then subjected to western blot analysis using antibodies against phospho-mTOR (p-mTor; at Ser2481), phospho-Akt (p-Akt; at Thr308) or β-actin. One representative experiment out of n = 3 is shown. **(B)** Cells were cultured as in **(A)** and counted after 72h of treatment. Results are presented as mean +/- SEM of n = 3 independent experiments and data was analyzed using the Student’s t-test, **p*<0.05 compared to the control (mIgG_1_ treated cells).

**SUPPLEMENTARY FIGURE 6: CD44 mAb treatment induces differentiation of HL60 and THP-1 cells but not KG1a cells. (A)** HL60, KG1a and THP-1 cells were treated with mIgG1 (white bars) or A3D8 (gray bars) for 72 h in an *in vitro* culture and flow cytometric analysis for the expression of the differentiation markers CD11b and CD15 were assessed. The bar graphs show that A3D8 treatment effectively induced the differentiation of HL60 and THP-1 but not KG1a cells.^[16](#_ENREF_16)^ Note that treatment of HL60 cells with rapamycin inhibited their proliferation but did not result in granulocytic differentiation (data not shown; and reference^[17](#_ENREF_17)^), suggesting that mTOR inhibition is not sufficient to induce differentiation of AML cells. These plots are representative of n = 4 independent experiments tracking the differentiation of cells following CD44 mAb treatment. **(B)** Analysis of differentiation of human cells treated *in vivo* with anti-CD44 antibody or IgG isotype control as described in Materials and Methods as well as **SUPPLEMENTARY Fig. 4**. CD45^+^ cells isolated from bone marrow of NOD/SCID mice Following treatment for 2 weeks, the human CD45^+^ (representative of HL60 cells) fraction of the bone marrow was stained with CD14 and CD15. The results presented are representative of n = 5 mice and show the increase in percentage of positive cells for the differentiation markers, CD14 and CD15. **(C)** Morphological analysis of HL60 and THP-1 cells using Giemsa staining. A3D8 treated cells showed decrease in nucleus:cytoplasm ratios, chromatin condensation, fewer nucleoli and irregular cytoplasm shape, which are characteristics of differentiated granulocytic cells.

**SUPPLEMENTARY VIDEO 1**: Z-stack rendering of control HL-60 cells stained with anti-FOXO3 conjugated to AlexaFluor 488 (green).

**SUPPLEMENTARY VIDEO 2**: Z-stack rendering of control HL-60 cells stained with anti-FOXO3 conjugated to AlexaFluor 488 (green). Signal was merged with DAPI-stained nuclei (blue).

**SUPPLEMENTARY VIDEO 3**: Three-dimensional reconstruction of the DAPI (blue) and FOXO3 (green) fluorescence signal in control HL-60 cells.

**SUPPLEMENTARY VIDEO 4**: Z-stack rendering of A3D8-treated HL-60 cells stained with anti-FOXO3 conjugated to AlexaFluor 488 (green).

**SUPPLEMENTARY VIDEO 5**: Z-stack rendering of A3D8-treated HL-60 cells stained with anti-FOXO3 conjugated to AlexaFluor 488 (green). Signal was merged with DAPI-stained nuclei (blue).

**SUPPLEMENTARY VIDEO 6**: Three-dimensional reconstruction of the DAPI (blue) and FOXO3 (green) fluorescence signal in A3D8-treated HL-60 cells.

**SUPPLEMENTARY VIDEO 7**: Z-stack rendering of control HL-60 cells stained with DAPI (blue).

**SUPPLEMENTARY VIDEO 8**: Z-Stack rendering of A3D8-treated HL-60 cells stained with DAPI (blue).

**SUPPLEMENTARY REFERENCES**

1. Dalton WT, Jr., Ahearn MJ, McCredie KB, Freireich EJ, Stass SA, Trujillo JM. HL-60 cell line was derived from a patient with FAB-M2 and not FAB-M3. *Blood* 1988 Jan; **71**(1)**:** 242-247.

2. Vardiman JW, Thiele J, Arber DA, Brunning RD, Borowitz MJ, Porwit A*, et al.* The 2008 revision of the World Health Organization (WHO) classification of myeloid neoplasms and acute leukemia: rationale and important changes. *Blood* 2009 Jul 30; **114**(5)**:** 937-951.

3. Odero MD, Zeleznik-Le NJ, Chinwalla V, Rowley JD. Cytogenetic and molecular analysis of the acute monocytic leukemia cell line THP-1 with an MLL-AF9 translocation. *Genes Chromosomes Cancer* 2000 Dec; **29**(4)**:** 333-338.

4. Morgan MA, Wegner J, Aydilek E, Ganser A, Reuter CW. Synergistic cytotoxic effects in myeloid leukemia cells upon cotreatment with farnesyltransferase and geranylgeranyl transferase-I inhibitors. *Leukemia* 2003 Aug; **17**(8)**:** 1508-1520.

5. Xu Q, Thompson JE, Carroll M. mTOR regulates cell survival after etoposide treatment in primary AML cells. *Blood* 2005 Dec 15; **106**(13)**:** 4261-4268.

6. Chapuis N, Tamburini J, Green AS, Vignon C, Bardet V, Neyret A*, et al.* Dual inhibition of PI3K and mTORC1/2 signaling by NVP-BEZ235 as a new therapeutic strategy for acute myeloid leukemia. *Clin Cancer Res* 2010 Nov 15; **16**(22)**:** 5424-5435.

7. Nishioka C, Ikezoe T, Yang J, Gery S, Koeffler HP, Yokoyama A. Inhibition of mammalian target of rapamycin signaling potentiates the effects of all-trans retinoic acid to induce growth arrest and differentiation of human acute myelogenous leukemia cells. *Int J Cancer* 2009 Oct 1; **125**(7)**:** 1710-1720.

8. Nishioka C, Ikezoe T, Yang J, Koeffler HP, Yokoyama A. Blockade of mTOR signaling potentiates the ability of histone deacetylase inhibitor to induce growth arrest and differentiation of acute myelogenous leukemia cells. *Leukemia* 2008 Dec; **22**(12)**:** 2159-2168.

9. Gadhoum Z, Leibovitch MP, Qi J, Dumenil D, Durand L, Leibovitch S*, et al.* CD44: a new means to inhibit acute myeloid leukemia cell proliferation via p27Kip1. *Blood* 2004 Feb 1; **103**(3)**:** 1059-1068.

10. AbuSamra DB, Al-Kilani A, Hamdan SM, Sakashita K, Gadhoum SZ, Merzaban JS. Quantitative characterization of E-selectin interaction with native CD44 and PSGL-1 using a real-time immunoprecipitation-based binding assay. *The Journal of biological chemistry* 2015 Jun 29.

11. Chapuis N, Park S, Leotoing L, Tamburini J, Verdier F, Bardet V*, et al.* IkappaB kinase overcomes PI3K/Akt and ERK/MAPK to control FOXO3a activity in acute myeloid leukemia. *Blood* 2010 Nov 18; **116**(20)**:** 4240-4250.

12. Santamaria CM, Chillon MC, Garcia-Sanz R, Perez C, Caballero MD, Ramos F*, et al.* High FOXO3a expression is associated with a poorer prognosis in AML with normal cytogenetics. *Leukemia Res* 2009 Dec; **33**(12)**:** 1706-1709.

13. Saland E, Boutzen H, Castellano R, Pouyet L, Griessinger E, Larrue C*, et al.* A robust and rapid xenograft model to assess efficacy of chemotherapeutic agents for human acute myeloid leukemia. *Blood Cancer J* 2015; **5:** e297.

14. Jin L, Hope KJ, Zhai Q, Smadja-Joffe F, Dick JE. Targeting of CD44 eradicates human acute myeloid leukemic stem cells. *Nat Med* 2006 Oct; **12**(10)**:** 1167-1174.

15. Zoller M. CD44: can a cancer-initiating cell profit from an abundantly expressed molecule? *Nat Rev Cancer* 2011 Apr; **11**(4)**:** 254-267.

16. Charrad RS, Gadhoum Z, Qi J, Glachant A, Allouche M, Jasmin C*, et al.* Effects of anti-CD44 monoclonal antibodies on differentiation and apoptosis of human myeloid leukemia cell lines. *Blood* 2002 Jan 1; **99**(1)**:** 290-299.

17. Xie N, Zhong L, Liu L, Fang Y, Qi X, Cao J*, et al.* Autophagy contributes to dasatinib-induced myeloid differentiation of human acute myeloid leukemia cells. *Biochemical pharmacology* 2014 May 1; **89**(1)**:** 74-85.
