# Supplementary figures and images for: Anti-CD44 antibodies inhibit both mTORC1 and mTORC2: a new rationale supporting CD44-induced AML differentiation therapy
Source: Leukemia. 2016 Sep 13;30(12):2397–401. doi: 10.1038/leu.2016.221 (PMC5155032; doi:10.1038/leu.2016.221)

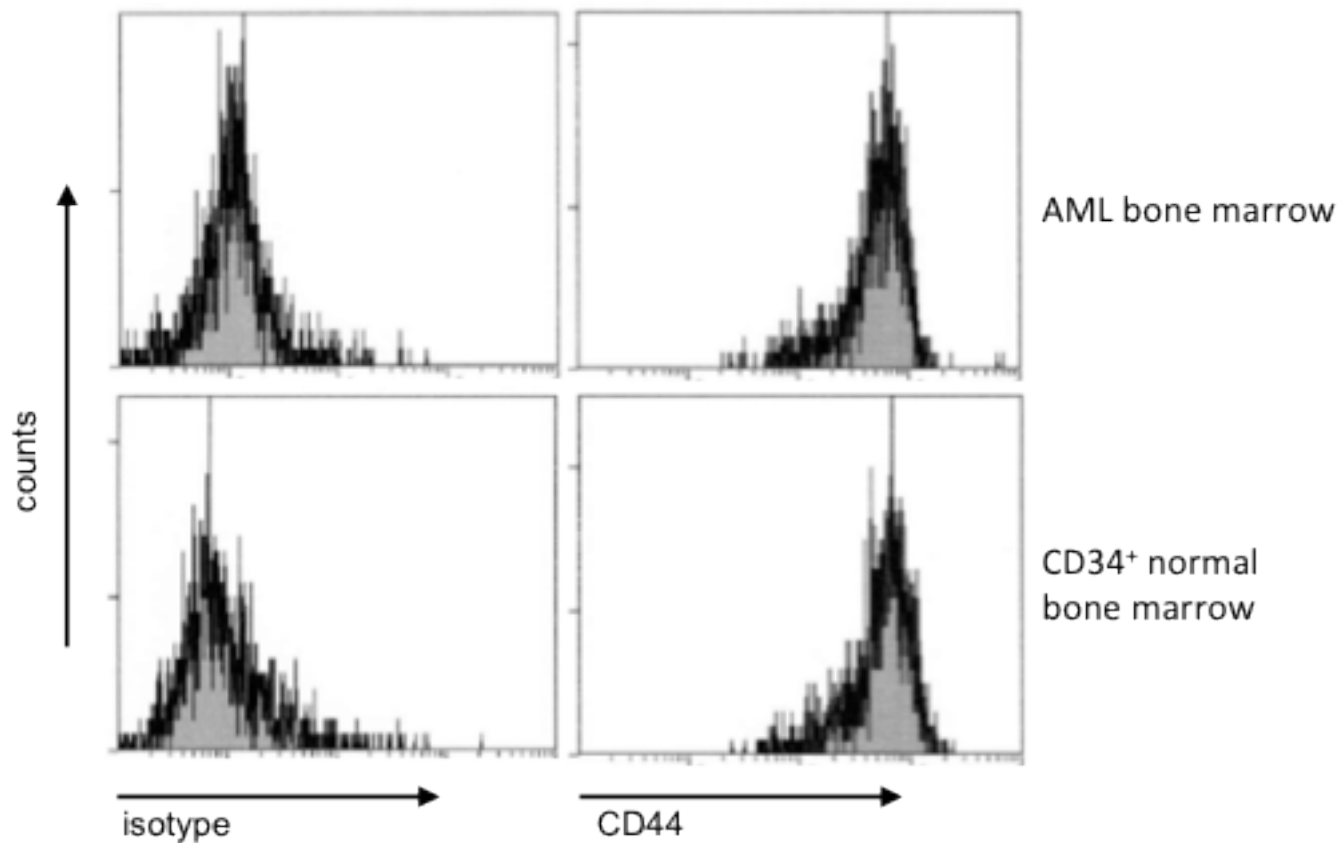

Supplementary Figure 1

Supplement: Supplementary Figure 1 [file leu2016221x2.pdf]

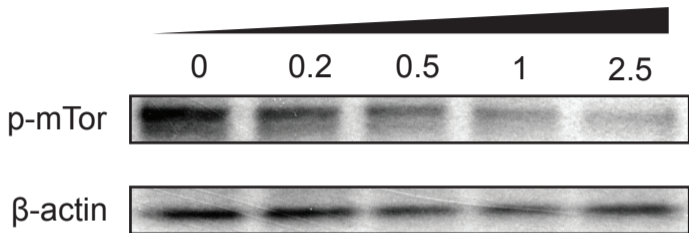

Supplementary Figure 2

Supplement: Supplementary Figure 2 [file leu2016221x3.pdf]

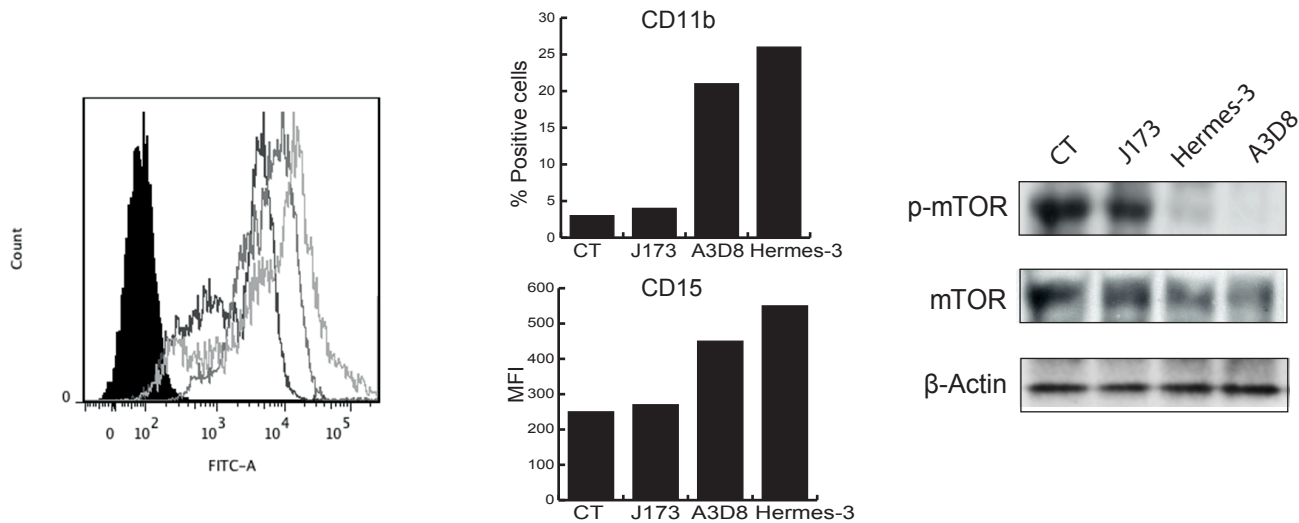

Supplementary Figure 3

Supplement: Supplementary Figure 3 [file leu2016221x4.pdf]

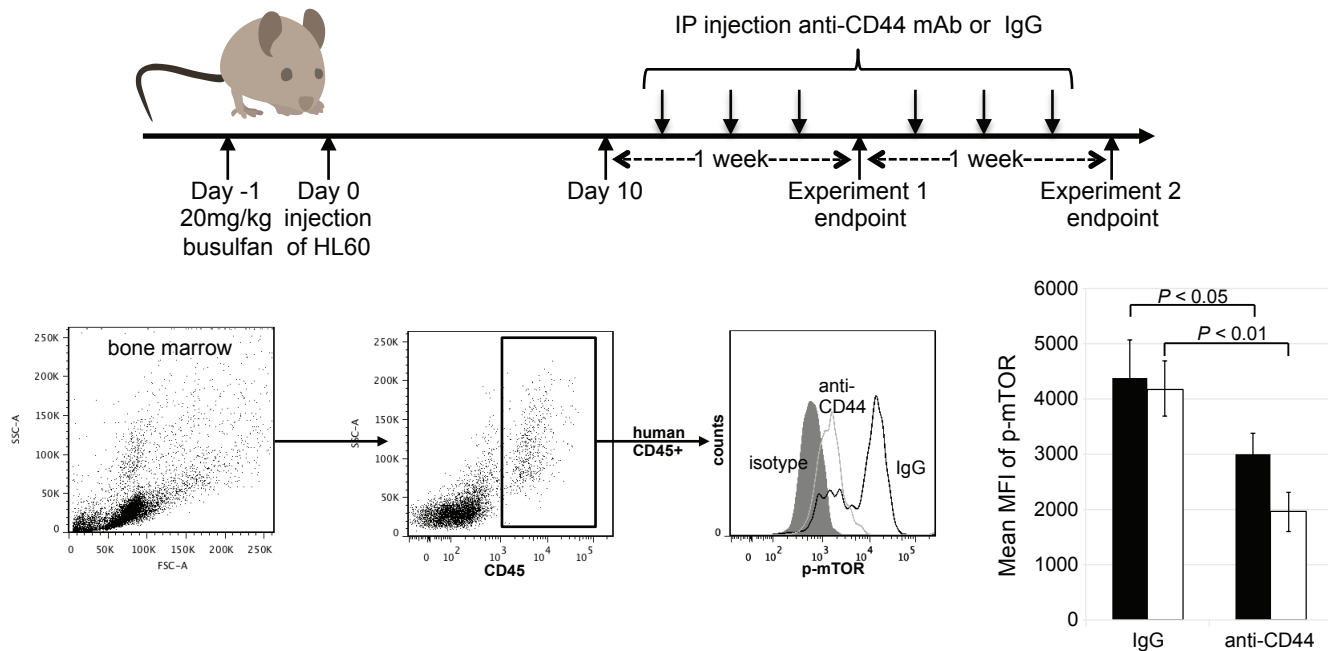

Supplementary Figure 4

Supplement: Supplementary Figure 4 [file leu2016221x5.pdf]

A

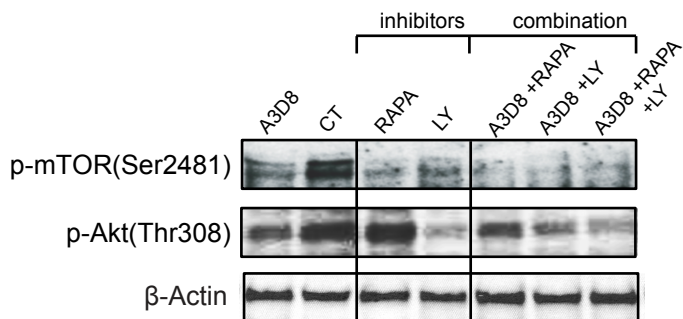

B

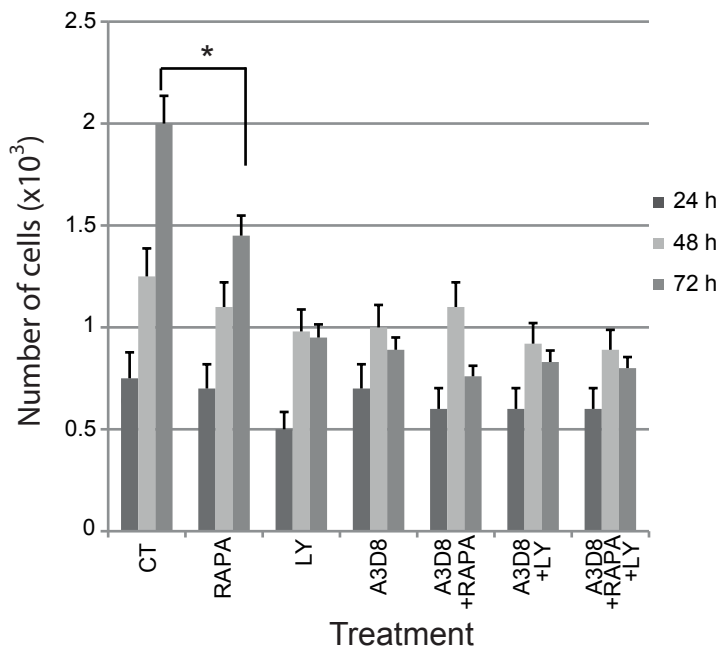

Supplementary Figure 5

Supplement: Supplementary Figure 5 [file leu2016221x6.pdf]

A

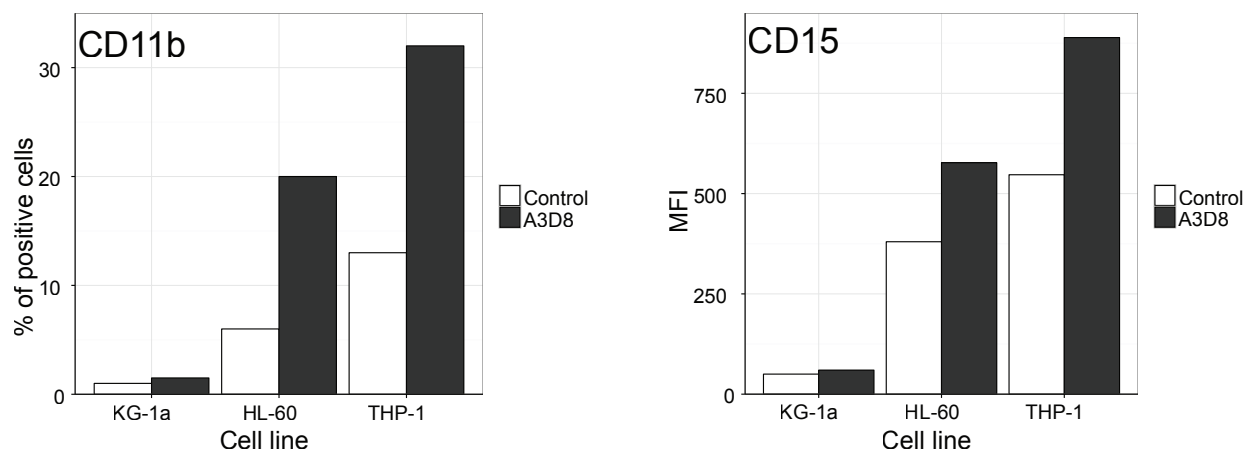

B

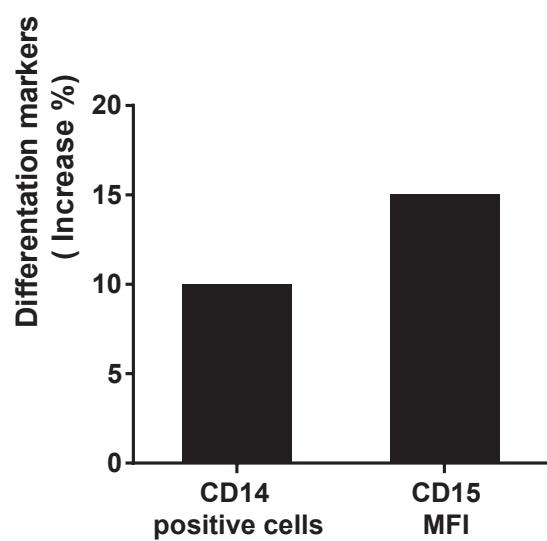

C

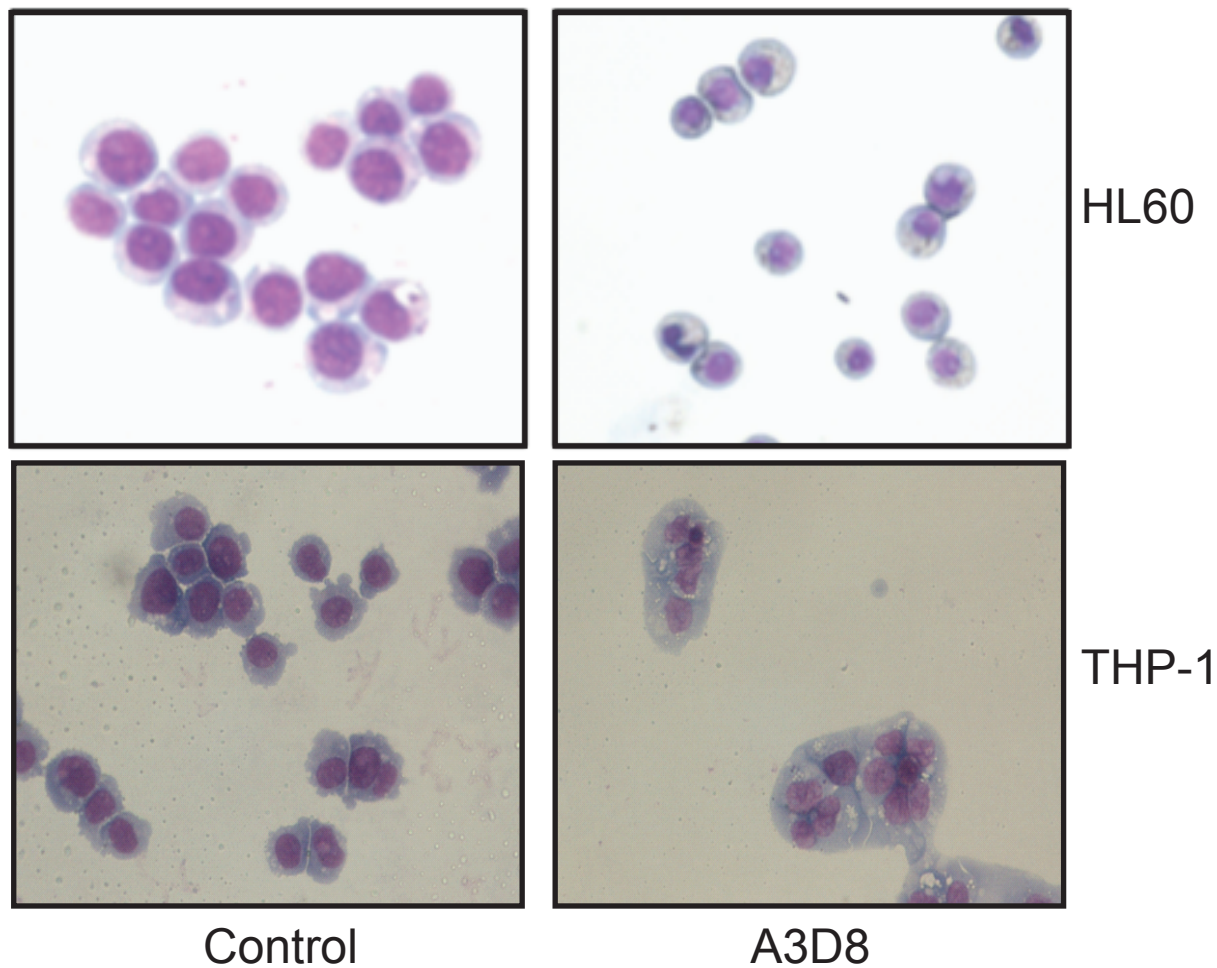

Supplementary Figure 6

Supplement: Supplementary Figure 6 [file leu2016221x7.pdf]
